# Chondrocyte De-Differentiation: Biophysical Cues to Nuclear Alterations

**DOI:** 10.3390/cells11244011

**Published:** 2022-12-12

**Authors:** Noor A. Al-Maslamani, Rachel Oldershaw, Simon Tew, Jude Curran, Pieter D’Hooghe, Kazuhiro Yamamoto, Henning F. Horn

**Affiliations:** 1 Department of Musculoskeletal and Ageing Science, Institute of Life Course and Medical Sciences, University of Liverpool, Liverpool L7 8TX, UK; 2 Department of Mechanical, Materials and Aerospace Engineering, School of Engineering, University of Liverpool, Liverpool L69 3GH, UK; 3 Department of Orthopaedic Surgery, Aspetar Orthopaedic and Sports Medicine Hospital, Doha P.O. Box 29222, Qatar; 4 College of Health and Life Sciences, Hamad Bin Khalifa University, Doha P.O. Box 34110, Qatar

**Keywords:** autologous chondrocyte implantation, de-differentiation, re-differentiation, chondrocyte, mechanobiology, biophysics, two-dimensional culture (2D), three-dimensional culture (3D)

## Abstract

Autologous chondrocyte implantation (ACI) is a cell therapy to repair cartilage defects. In ACI a biopsy is taken from a non-load bearing area of the knee and expanded in-vitro. The expansion process provides the benefit of generating a large number of cells required for implantation; however, during the expansion these cells de-differentiate and lose their chondrocyte phenotype. In this review we focus on examining the de-differentiation phenotype from a mechanobiology and biophysical perspective, highlighting some of the nuclear mechanics and chromatin changes in chondrocytes seen during the expansion process and how this relates to the gene expression profile. We propose that manipulating chondrocyte nuclear architecture and chromatin organization will highlight mechanisms that will help to preserve the chondrocyte phenotype.

## 1. Introduction

Cells in the human body experience a variety of mechanical forces. The location of the cells determines what type of forces the cells are exposed to: e.g. cardiomyocytes experience tensile forces by cardiac contractions [1], endothelial cells are exposed to shear forces by fluid flow [2] and chondrocytes reside in an environment that is exposed to compressive, tensile and shear forces [3,4,5].

To examine how cells respond to mechanical forces, researchers have used different approaches, including cell stretchers, growing cells on substrates of difference stiffness, and embedding cells in hydrogels of ranging stiffness. However, less attention is given to how cells from different environments adapt to these artificial culturing environments and how this alters the spatial chromatin architecture and organization. Indeed, the chromatin spatial organization of the liver is different from that of the heart and this organization directly contributes to cell-specific transcriptome [6]. Chromatin compartmentalization is an inherent property of the nuclear architecture, with euchromatin being more accessible to transcriptional factors and heterochromatin less accessible [6,7]. In the heart, cardiac-specific genes are found in areas of euchromatin, while liver-specific genes are found in heterochromatin areas [6]. This illustrates that the packaging of the genome is not random and has direct implications on gene expression profiles. However, to date we do not fully understand how culturing cells in different mechanical environments alters the gene organization and compartmentalization.

Monolayer culture on plastic has been the traditional cell expansion method for cell therapies such as autologous chondrocyte implantation (ACI). Chondrocytes, which normally have a rounded morphology in their native tissue niche adopt an elongated fibroblastic-like morphology on plastic, leading to intracellular and intra-nuclear changes that ultimately result in a loss of chondrocyte phenotype (de-differentiation). On the other hand, culturing chondrocytes in 3D culture systems appears to maintain and/or re-differentiate the chondrocyte phenotype [8]. The main differences between monolayer and three-dimensional cultures are the geometrical constraints impinged on the chondrocyte. The adaptation of chondrocytes to these different constraints leads to not only cytoskeletal changes, but clear alterations in nuclear shape and DNA conformation and organization [9,10].

The de-differentiation phenomenon is complex; in this review, we aim to focus on this phenomenon in-vitro and dissect the biophysical aspects affecting nuclear architectural changes implicating phenotypic changes. We will provide a brief overview of the chondrocyte microenvironment, ACI expansion methodology and the de-differentiation observed in monolayer expansion. We will examine this de-differentiation from mechanobiology and biophysics perspectives and highlight effects on chromatin organization and gene expression. We discuss various biophysical aspects that could influence de-differentiation and suggest that by manipulating chondrocytes mechanically we can further dissect the de-differentiation phenomenon and find ways to preserve the chondrocyte phenotype.

## 2. Chondrocytes in Their Native Niche: Healthy and Diseased

Chondrocytes are responsible for building hyaline cartilage [11], a tissue which is able to withstand substantial mechanical forces [11,12] and is essential for healthy joints. Chondrocytes reside in an avascular, aneural and anaerobic environment [11,13]. They are nourished via mechanically stimulated diffusion of nutrients through the extracellular matrix. Consequently, mechanical stimulation dynamics influence chondrocyte survival, and lack of mechanical stimulation prevents nutrient diffusion to the chondrocytes, leading to cell death [12].

The main components of cartilage tissue are water, fibrillar and non-fibrillar collagen, and negatively charged proteoglycans [14]. This composition provides cartilage with viscoelastic properties, a biphasic nature (fluid/solid) under mechanical strain, and a recovery phase after mechanical loading [15]. Cartilage loading causes water squeezes out and a force transmission to the cell and its nucleus [14]. Clinical in-vivo studies using magnetic imaging have given novel insights into the viscoelastic behaviour of cartilage in daily activity [15]. For example, after performing 50 knee bends, the cartilage volume decreased by 5%, and after 45 min of rest, only 50% of the volume was recovered [16]. In another study, 100 knee bends required more than 90 min to recover full cartilage height [17]. While, mechanical stimulation is essential for chondrocyte survival, excessive loading can lead to tissue injury, tissue remodelling, and gradual deterioration of the tissue [12]. Cartilage deterioration can also be induced by reduced mechanical mobility: joint immobilization leads to a loss of proteoglycan content in the knee. In a joint immobilization study the right knee in dogs was immobilized in flexion (90°) for 11 weeks [18]. The immobilization caused a general reduction of proteoglycan content at all cartilage surfaces sites and distinctively presented a clear 64% reduction in proteoglycan at the periphery of the femoral condyles [18]. When these dogs were remobilized, the proteoglycan content remained 18% lower level than the aged matched controls, even after 15 weeks [18]. These studies signify the importance of mechanics for cartilage tissue preservation and chondrocyte survival.

The cartilage tissue is subdivided into four zone: superficial zone, middle zone, deep zone, and calcified cartilage. Within the superficial zone, chondrocytes have an elongated morphology parallel to the articulating surface, compared to the middle zone where they are rounded, and the deep zone, where they have a columnar arrangement of enlarged cells perpendicular to the articulating surface. In addition to cell morphological differences, alignment of collagen fibrils and proteoglycan content is also zone dependent. In the superficial zone, the collagen fibers are aligned parallel to the articulating surface, compared to the deep zone where the collagen fibers are aligned perpendicular [19]. The proteoglycan content is lower in the superficial zone compared to the deep zone [20]. Additionally, elastin is found extensively aligned with collagen fiber parallel to articulating surface in the superficial zone, providing this zone with elastic properties [21,22,23].

The fate of the chondrocyte is dictated by interactions with the extracellular matrix. Chondrocytes in their native niche are surrounded by a 2 to 4-micron pericellular matrix (PCM), which together with the cell is termed the chondron. The PCM is a crucial mechanical transducer to the chondrocyte [15,24], as it provides the chondrocyte with enhanced mechanical properties. The PCM of the chondrocyte at superficial zone differs from that of the deep zone in that the superficial zone is enriched in elastin and lipids while the deep zone showed no presence of lipids nor elastin [23,25]. The PCM composition significantly dictates the mechanical forces that are transmitted to the chondrocyte [26]. In cartilage explant compression studies, the strain on the chondrocyte vs. the PCM was investigated under different compression forces: 10%, 30% and 50%. These studies found that at these compression levels the PCM in the superficial zone absorbs most of the applied force, shielding the chondrocyte [26]. In contrast, chondrocytes from the deep and middle zones experienced most of the strain at 10% and 30% compressions [26]. At compressions of 50%, the PCM in all three zones absorbs most of the force [26]. These findings suggest that the PCM serves as an amplifier or dampener of force to the chondrocyte, depending on the level of force applied.

The PCM within the superficial zone is much stiffer (40–100 kPa) than the PCM in the middle and deep zones (24–60 kPa). The chondrocyte in the superficial zone are also stiffer (0.55 kPa) than in the middle and deep zones of cartilage (0.29 kPa) [27,28,29]. This suggests that the presence of the PCM is critical to the mechanical stability of chondrocytes. In chondron/chondrocyte compression studies, chondrons displayed a higher stiffness and required higher forces to rupture than an isolated chondrocytes [30]. The presence of the PCM lowers the grade of the force that is transmitted to the chondrocyte, providing the chondrocytes with a protective barrier and higher probability to survive under mechanical loading, as most of the force is taken by the pericellular network surrounding the cell [30]. Under mechanical loading, the PCM also filters the nutrients reaching the chondrocyte, as these molecules must diffuse through the PCM mesh to reach the chondrocyte [31]. Thus, the PCM serves both as a transducer and a filter of biomechanical and biochemical signals to the chondrocyte.

The PCM is composed of collagen types II, VI, and IX, aggrecan, hyaluronan, decorin, fibronectin, and characterized by the presence of smaller collagen fibers and high concentration of proteoglycans relative to other regions of the ECM [32,33]. Changes to this composition promote degenerative diseases such as osteoarthritis (OA). The onset of OA is initially reflected in the PCM, where chondrocytes remodel the composition to decrease aggrecan content while increasing expression of collagen type X, matrix metalloproteinases (MMP1 and MMP13), and various ADAMTS (a disintegrin and metalloproteinase with a thrombospondin motifs)—ADAMTS 1,4,5 [34]. MMP13 and ADAMTS5 are highly expressed and active in degrading the collagen and aggrecan, respectively [34,35]. These changes initially alter the PCM, then the ECM, and gradually affect the mechanical integrity of the tissue. The activities of matrix degrading proteins is intensified by high levels of nitric oxide, a molecule that is upregulated by inflammatory proteins, such as IL-1β [34]. IL-1β, downregulates matrix synthesis and up-regulates metalloprotease synthesis via nitric oxide (NO), leading to an increase in matrix degradation and chondrocyte apoptosis [34,35].

In healthy cartilage, the low-density lipoprotein receptor-related protein 1 (LRP1), also known as CD91, is a cell-surface protein which maintains cartilage homeostasis by clearing matrix degrading proteins (MMP13, ADAMTS4,5) through endocytosis [36,37]. In OA, LRP1 is impaired because of shedding by MMP14 and ADAM17 [36]. This combination of upregulation of matrix-degrading proteins and downregulation of ECM protection promotes the loss of hyaline cartilage. Therefore, understanding the expression of molecules such as LRP1 during the de-differentiation and re-differentiation process is central to the success of the ACI procedure.

## 3. The ACI Procedure

**Figure 1:**
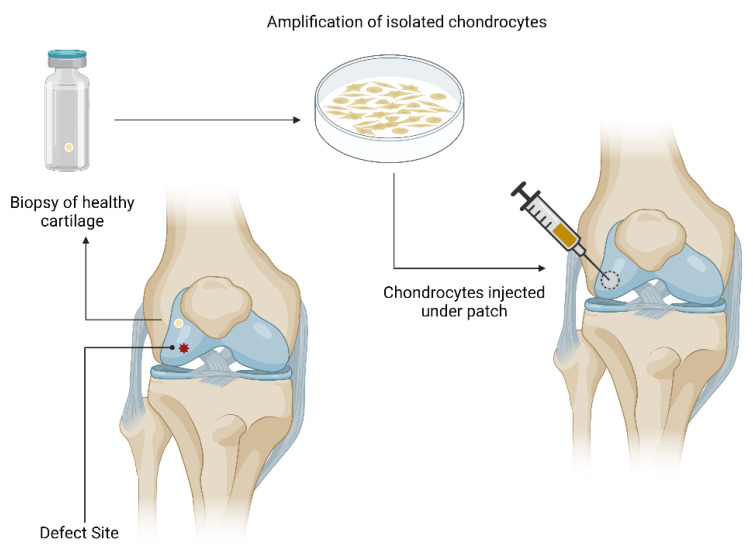
Autologous Chondrocyte Implantation procedure. Damaged cartilage is depicted in red. A tissue biopsy is taken from a healthy sight of the knee (non-load baring area). The cells are then expanded in-vitro and then injected back into the defect sight, where it is sutured with patch (periosteal or biomembrane). Created with BioRender.com (accessed on 23 November 2022).

Autologous chondrocyte implantation (ACI) is a two-stage operative procedure for the treatment of medium to large full thickness defects (2–10 cm^2^) in cartilage (Figure 1) [38]. The first generation ACI was first introduced in 1987 by Grande et al. [39] and clinically applied in 1994 by Brittberg et al. [40]. In these first procedures, cartilage slices were harvested arthroscopically from a healthy non-weight-bearing region of the cartilage [19,21]. Chondrocytes were released from the cartilage tissue through enzymatic digestion and expanded in monolayer (25 cm^2^ or 75 cm^2^ culture flasks) over a period of 11–21 days to yield approximately 2.6 to 5 million cells [38,40,41]. The cultured chondrocytes were then implanted into the affected area and patched using a periosteal patch [42,43]. Theoretically, the periosteal patch should provide the cells with growth factors that encourage chondrocyte development and differentiation [42,43]. However, the periosteal patch was found to cause significant graft hypertrophy. This has encouraged researchers to investigate other biomaterials such as collagen patches [42,43]. One such patch is collagen type I and III that is currently being marketed as Chondro-Gide [42,44].

An important disadvantage associated with ACI is the accessibility to high cell numbers. Since these cells are harvested from patients via a small tissue biopsy procedure, the number of chondrocytes isolated from native cartilage is limited. However, large quantities of chondrocytes are needed for cartilage repair, which makes ex-vivo expansion essential for any potential use in ACI. When chondrocytes are expanded, they are cultured in a monolayer 2D culturing system, which is an effective method that generates a large number of viable cells [45]. Several commercial ACI expansion companies use two-dimensional monolayer systems to provide patients with 4 to 12 million viable cells for implantation (Table 1). A recent focus in ACI is on priming the chondrocyte phenotype through the transfer of the chondrocytes to a 3D matrix [46] that is supplemented with biomolecules. To promote the hyaline forming potential of chondrocytes, current biomolecule cocktails include L-ascorbic acid-2-phosphate, dexamethasone, transforming growth factor β3 (TGF-β3) and insulin-transferrin-selenium (ITS) [45]. However, the outcome of this procedure is still inconsistent and a variety of tissues are formed: fibrous, fibrous-hyaline, and hyaline cartilage.

## 4. Chondrocytes Expansion and the De-Differentiation Effect

At a cellular level, 2D expansion on tissue culture plastic causes chondrocytes to gradually adapt to the monolayer environment by de-differentiating and losing the chondrocyte phenotype. De-differentiation is a process by which a cell goes from a terminally or partially differentiated stage to a less differentiated stage within their own lineage [47]. This phenomenon is manifested by a change in cell shape, gene and protein expression and importantly, cellular function [47].

The de-differentiation phenomenon is also observed in isolated chondrons grown in monolayer. When chondrons are cultured over a period of 7 days, two populations are observed: a floating population of chondrocytes that has a PCM, and a population of chondrocytes that has lost its PCM and adheres to tissue culture plastic [48]. Gene expression profiles of these different populations show the floating population has significantly higher *SOX9*, collagen type II, aggrecan, *COMP*, collagen type X, and lower *RUNX2*, compared to chondrocytes that adhered to the surface [48]. These changes highlight the adaptation chondrocytes go through to adhere to tissue culture plastic and that the PCM geometric constraints plays a crucial role in maintaining the chondrocyte phenotype. Only after the loss of the PCM do chondrocytes adhere to the culturing surface [48], presenting de-differentiation as a process of adaptation to a new environment.

When chondrocytes without their PCM are isolated and expanded on 2D plastic, they change from spherical-shaped chondrocytes to spindle-shaped, fibroblastic-like cells. In addition, collagen type II, the major collagen produced by chondrocytes is switched to the production of type I collagen [49] along with a decrease in aggrecan, collagen type XI, and collaged type IX [8,50,51,52]. These cells switch from the secretion of a hyaline cartilage ECM to a fibrous cartilage ECM, a matrix that is mechanically inferior.

Recent studies have compared the de-differentiation of chondrocytes and their loss of functional in-vitro status to degenerative diseases, such as OA. With the onset of OA, cartilage is degraded and the chondrocytes undergoes a panel of changes, including phenotypic changes. In situ staining of OA cartilage, identified three chondrocyte phenotypes: activated, hypertrophic and fibrotic [49]. The de-differentiation-like phenotype that is observed in OA is reflected by a population chondrocytes within the upper middle zone where a shift from quiescent to proliferative state is observed, with a deposition of fibrotic markers such as collagen type I and III [49,53,54]. While middle zone chondrocytes, presented with an activated phenotype, chondrocytes that are producing collagen type II, and the deep zone chondrocytes were hypertrophic, participated in matrix calcification and degradation via the expression of collagen type X and MMP13 [49,55]. These zonal differences emphasize and suggest the adaptation of chondrocytes to the environmental change is highly dependent on their innate environmental differences.

This de-differentiation phenotype is a major challenge for cell-based therapies such as ACI [56,57,58].These findings suggest that if de-differentiated cells were implanted they would cause a catabolic effect [52,59]. This has driven much of the research to understand the de-differentiation phenotype and its regenerative and degenerative potential for a procedure such as ACI.

## 5. Chondrocytes in Two and Three-Dimensional Culture Compared to Native Tissue

In 2016 A.J. Mueller et al. investigated the transcriptional profile of in-vitro culturing systems both monolayer and three-dimensional systems compared to native cartilage. They found adult cartilage tissue is characterized by the expression of collagen type II, and aggrecan [59] as also shown by other studies. Proteoglycans (aggrecan, proteoglycan 2 and 3), tubulins, actin nucleator (*Wasp*, *Arpc5 and Actr2*) and kinesins (*Kif4a*, *Kif11*, *Kif15*, *Kif20a/b*, *Kif22*, *Kif23*) are strongly represented in cartilage comparative to monolayer chondrocytes [59]. On the other hand, actin assembling units, profilin-2 and cofilin-2, were downregulated in cartilage comparative to monolayer chondrocytes [59]. By passage five of 2D expansion, cells exhibited high expression levels of developmental mesenchymal markers: *Thy1* (CD90), epithelial-mesenchymal transition regulator *Snai1*, prion protein encoding gene, *Prnp*, and bHLH transcription factor *Twist1* [59]. Thus, monolayer expansion results in significant changes in cellular expression profile with a clear shift towards mesenchymal pre-cursor cell lineage.

When comparing cartilage to chondrocytes grown in 3D culture (alginate), 3D culture caused an overexpression of AP-1 (*Fos* and *Junb*), the transmembrane glycoprotein osteoactivin gene *Gpnmb*, clusterin and the bone morphogenetic protein receptor type 1a (*Bmpr1a*) [59]. 3D culture was also found to upregulate genes associated with oxidative stress (*Nfe2l2*), hypoxia (*Hif1a*) and antioxidant responses (*Sod2*, *Hmox1*) [59]. It is therefore clear that while both culture types do not mimic the expression profile of native tissue, the 3D culture appears to have fewer changes than those seen in 2D culture. However, the major limitation in 3D culture, is cellular proliferation is very low [60]. This makes 3D culture a preferred method to re-differentiation chondrocytes after monolayer expansion [60].

## 6. The Biophysical Aspects of Monolayer Expansion and De-Differentiation

Chondrocytes are mechanoresponsive cells that reside in a mechanically active tissue. In the process of ex-vivo expansion, these cells are harvested from a 3D, mechanically active niche and moved onto a 2D, non-mechanically active environment. During this process, chondrocytes adapt to these physical changes and begin exhibiting an elongated fibroblastic-like phenotype with elongated nuclei. A change in nuclear morphology is often observed in tissues that are mechanically stimulated as cartilage sections (discs). Static compressions on 3 mm cartilage discs, showed chondrocytes starting with a round morphology and ending with an elongated shape at high static compressions [61]. At a single cell level, static compressions have shown that increased mechanical force exposure is experienced by the nucleus, which undergoes considerable change in both structure and volume [62]. This nuclear change leads to a shift in single-cell mRNA expression to a more catabolic state, with decreased expression of aggrecan, collagen type IIA, and an increase in tissue inhibitor of metalloproteinase-1 (*TIMP1*) in response to 60 s of applied force [62]. These findings emphasize that changes in nuclear properties impact gene expression through alterations in chromatin folding and translocation of transcriptional factors [63,64]. In monolayer culture, chondrocytes gain a fibroblastic like phenotype with an elongated nucleus to adapt to these physical changes for the duration of expansion, which could range up to a couple of weeks. This prolonged culture leads to nuclear alterations that have permanent and irreversible consequence on the expression profile.

### 6.1. Integrin Profile Changes during Expansion

The process of mechanical sensing starts at the cell periphery, where the cell forms a physical connection with its environment [65]. Integrins are the main force transducer between the environment and the cell and serve as mechanical linkers between the cytoskeleton and the environment [65,66,67,68]. A family of transmembrane proteins that sit in the plasma membrane, integrins are heterodimers composed of α and β subunits. The bulk of these proteins is found in the extracellular domain with 700 aa of the α subunit and 1000 aa of β subunit protruding into the extracellular space [65,69]. By contrast, the cytoplasmic tail is 40–70 amino acids long. Integrins are maintained in a bent conformation when inactive. The activation process causes conformational changes within the cytoplasmic domain, where the protein talin binds to the β subunit and triggers the activation and conformational change of the α and β subunits [69]. Upon activation, the extracellular domains of integrins bind ECM protein such as fibronectin, collagen and others [68,70]. In the cytoplasm, the β subunit of the integrin heterodimer binds to the actin cytoskeleton through a variety of adaptor proteins. As the ECM ligand binds, the integrins activates further and clusters to initiate the assembly of the focal adhesions (FA) complex that is composed of focal adhesions kinase (FAK), vinculin, paxillin, and tensin, thus forming a linkage between the cell and the environment [68,70]. In stiff matrices there are increased number of focal adhesions and traction forces generated between FA and ECM, as compared to soft matrices that have fewer focal adhesions [68,70]. In addition, FA complexes contribute to the reorganization of the actin cytoskeleton in response to mechanical stimuli [66,67] thus translating the stimuli from the extracellular environment into a cytoskeletal change.

Immunophenotyping performed on cartilage tissue showed the expression of α1β1 (collagen type VI, II and matrilin-1), α5β1 (fibronectin) and αVβ5 (fibronectin, vitronectin and osteopontin) and lesser amounts of α3β1 (fibronectin) and αvβ3 (COMP, fibronectin, vitronectin and osteopontin) [71,72,73]. The integrin profile is dependent on ECM proteins that are present. In OA, the ECM structure is altered, which leads to an alteration in integrin profile: α2β1 (collagen type II, VI, and chondroadherin), α4β1 (fibronectin and V-CAM) and α6β1 (laminin) integrins are the predominant integrins expressed in OA [73,74]. The changes in integrin profiles highlight the adaptation of chondrocyte to the ECM environment.

When chondrocytes are harvested for ACI, they are removed from an ECM-rich environment and cultured in an ECM-free environment. In the process of adaptation to an ECM free environment, chondrocytes de-differentiate and exhibit an integrin profile change. Primary chondrocytes cultured over a period of 21 days showed a clear increase in β1 and α2 levels by immunofluorescent analysis. This increase is consistent with the increase in α2β1 complex that is found in OA, suggesting that culturing chondrocytes for prolonged periods (21 days) on plastic can lead to a disease phenotype; however, this does not rule out the formation of other integrin complexes. Furthermore, it is clear evidence that chondrocytes sense and adapt to environmental changes by altering their integrin profile. Such changes will have downstream effects on cytoskeletal arrangement and tensions [75] as well as on nuclear responses [76].

### 6.2. Chondrocyte Nuclear Shape and Biomechanical Response to Substrate Rigidity

While mechanical sensing is mediated by integrins [77], the intracellular response is driven by the cytoskeleton. Rho GTPases (RhoA, Rac1 and Cdc42) are known as master regulators of actin cytoskeleton dynamics through actin nucleation (WASP/WAVE) and Diaphanous-related formins, affecting cell morphology and cell adhesion [78]. Cell morphology and adhesion are two aspects that are influenced by substrate rigidity. On stiff substrates, cells assemble stress fibers that induce high intracellular tension forces, while soft substrates do not promote stress fiber formation [79,80]. The cytoskeletal forces, mediated in part by these stress fibers, are transduced from the cytoskeleton to the nucleus through the LINC complexes (Linker of Nucleoskeleton to Cytoskeleton) [79,80].

Several studies have shown the impact of substrate stiffness on chondrocyte de-differentiation [81,82,83,84]. Q. Zhang et al. [81] investigated the effects of growing chondrocytes on a range of polydimethylsiloxane (PDMS) membranes stiffnesses: soft to stiff (stiff being similar to commercial petri dish). They showed that 78% of the cells grown on soft substrates exhibited and maintained a round chondrocyte morphology, while on stiffer substrates only 41% of the cells presented with a spherical morphology, with 59% having a stretched fibroblastic morphology [81]. E. Schuh et al. found that stiffer substrates lead to higher proliferation rates but that stiff substrates also led to phenotypic changes associated with low collagen type II and aggrecan expression, and high collagen type I expression [82]. By contrast, softer substrates promoted the maintenance of the chondrogenic phenotype with high collagen type II and aggrecan expression, and lower collagen type I expression [82]. Chondrocytes grown on different substrate rigidities also showed apparent differences in F-actin distributions [83] and actin depolarization has been shown to enhance the chondrogenic potential [85,86], while the loss of chondrocyte phenotype correlates with increased RhoA signaling and the presence of stress fibers [83,84]. On 54–135 kPa substrates, chondrocytes presented highly organized parallel stress fibers, with a wide spread polygonal morphology [83]. By contrast, on 1.4–6 kPa substrates chondrocytes had a much smaller and rounded morphology with actin filament extensions found only in few cells [83]. Because actin filaments are able to transmit force to the nucleus, substrate stiffness has been shown to contribute to lineage determination, and affect expression of NE proteins, including Lamin A/C [87,88].

### 6.3. Nuclear Lamins, Hetrochromatin and Euchromatin

Lamins are intermediate filament proteins that reside primarily within the internal periphery of the nucleus. Lamins are encoded by three genes: lamins A and C are alternative splice products of the *LMNA* gene, lamin B1 and lamin B2 are encoded by the *LMNB1* and *LMNB2* genes respectively [89]. Lamins are necessary to maintain nuclear structure and mechanical properties [90,91]. Lamins A/C primarily contribute to nuclear rigidity, while B-type lamins provide the nucleus with elastic properties [89,92]. Lamins have been shown to protect nuclear DNA against mechanical forces [93].

DNA in cells is generally found in one of two states. Heterochromatin is densely packed chromatin located at the periphery of the nucleus and is typically transcriptionally inactive [94]. Euchromatin on the other hand is gene rich with higher transcriptional activity and is located centrally with open structures [94]. The organization of chromatin is key to gene regulation and cell-fate determination [95]. Advances in microscopic imaging and molecular approaches have provided important insights into DNA localization and folding in normal versus disease states. The genome organization is an important player in regulating gene activity [96,97,98]. Lamins play an important role in chromatin organization [99], interacting with chromatin via lamina-associated domains (LADs) found mostly in heterochromatin. LADs are found in chromatin regions that contain mostly silent or weakly-expressed genes [100] and is enriched with repressive histone modifiers: H3K9me2, H3K9me3, and H3K27me3 [63]. Thus, the nuclear lamina helps to establish a repressive nuclear compartment at the nuclear periphery.

In the process of chondrocytes expansion for ACI, passage 0 (P0) chondrocytes have a rounded nucleus that is located in the center of the cell and expresses chondrocyte markers *COL2A* and *SOX9*. Using high resolution strain analysis to map mechanical strain on these chondrocytes, strain localization was distributed equally to heterochromatin and euchromatin at P0. At later passages, chondrocytes had a much flatter nucleus that was no longer centrally located in the cell. These later passage chondrocytes also had a higher strain in the heterochromatin and a higher expression of *COL1A1* [101]. Interestingly, late passage chondrocytes maintained *LMNB1* and *LMNB2* gene levels but had a significantly lower expression of lamin A/C, suggesting that a loss of nuclear structural integrity contributes to the expression of repressed genes. It is important to note, the loss of lamin A/C is also indicative of loss of resistive force around the nucleus periphery [92]. Similarly, Nava et al. showed the application of stain on progenitor cells leads to a decrease in nuclear envelope tension to prevent DNA damage. The reduction in tension is mediated by the reduction in H3K9me3 lamina-associated heterochromatin [63].

## 7. Conclusions: Mapping Nuclear Structural Changes to Understand De-Differentiation

De-differentiation is one of the main challenges for expanding chondrocytes in monolayer culture. By contrast, 3D cultures have been used as a method to re-differentiate these cells into chondrocytes. By providing chondrocytes with an added geometric dimension (3D vs. 2D), the cells are able to better retain their phenotype [59,60]. From a cellular biophysics perspective, the loss of chondrocytes 3D environment promotes chondrocyte stiffening through increased cortical actin, even within the first passage compared to freshly isolated chondrocytes [102]. This actin-driven changes in tension leads to downstream nuclear geometric changes. Hoshiba et al. demonstrated nuclear size differences between 3D (freshly isolated) and 2D (monolayer expanded) chondrocytes, and that a relationship exists between nuclear size and chondrogenic gene expression profiles [103]. They found freshly isolated chondrocytes have significantly smaller nuclei compared to passage 2 chondrocytes [103]. To demonstrate the relationship between nuclear changes and cartilaginous genes, they inhibited the polymerization of actin filament using cytochalasin D, in passage 2 chondrocytes that had larger nuclei [103]. They found the nuclear size of passage 2 chondrocytes significantly dropped and the expression level of aggrecan increased, while *SOX9* remained the same and collagen type I decreased [103]. How the mechanical and geometric environments lead to changes in cell expression is not fully understood. We know that the nucleus responds to some of these changes by altering nuclear mechanical attributes through changes in chromatin and the nuclear lamina [63,104,105]. In addition, lamin A/C regulates gene expression by modulating chromatin organization and the accessibility of transcriptional factors [91,106,107].

Studies by Nava et al. showed that applying cyclic uniaxial strain to epidermal progenitor cells for 30 min caused a differential expression of 480 genes, most of which are cell-cell junctions and cytoskeleton proteins. Longer applications of strain (up to six hours) caused an alteration in 2151 genes, with a strong upregulation in facultative heterochromatin marker H3K27me3 and its regulators (*JARID2* and *SUZ12*) [63]. In addition, downregulation of epidermal differentiation genes was found in these longer durations of strain. When application of cyclic uniaxial strain was extended beyond six hours (6–24 h) cells exhibited transcriptional repression and the irreversible silencing of differentiation genes [63]. In chondrocyte expansion, prolonged culture in a non-dynamic mechanical environment leads to the loss of chondrogenic potential that is irreversible [108,109]. Taken together, these findings suggest that prolonged exposure of cells to one type of environment (either dynamic or non-dynamic) can lock the cell into a physically non-reversible fate.

Recent studies have introduced the concept of mechanical memory, triggering lineage specification that are encoded within the nucleus structural changes and epigenetic plasticity from previous culturing environment [110,111]. Chromatin organization and acetylation can adapt rapidly to soft environments, which is reversible depending on time of exposure to a stiff environment. Scott et al. showed that monolayer expanded chondrocytes have a mechanical memory from their previous physical environment that induces epigenetic remodeling thereby impacting their fate and cellular performance in a later environment [111]. The repressive histone modifier H3K9me was observed to be near the nuclear envelope in freshly isolated chondrocytes; however, after passage 4 H3K9me3 foci were distributed evenly throughout the nucleus [111]. These chondrocytes were then introduced to a 3D culture to assess the intra-nuclear foci of H3K9me3 and their chondrogenic potential. After 10 days in 3D culture, H3K9me3 foci remained higher compared to freshly isolated chondrocytes in 3D. Also the passage 4 chondrocytes were able to rescue *SOX9* expression but not collagen type II and aggrecan [111]. Chondrocytes that were expanded for 2 passages, were able to regain their chondrogenic potential after 10 days in 3D culture, and their H3K9me3 foci decreased to a level comparable to freshly isolated chondrocytes in 3D [111]. This highlights the importance of understanding nuclear organization and biophysical adaptations during the chondrocyte expansion process. A better understanding of the chondrocyte nuclear mechanic tolerance is necessary to find methods to maintain or re-gain the chondrocyte phenotype.

**Figure 2:**
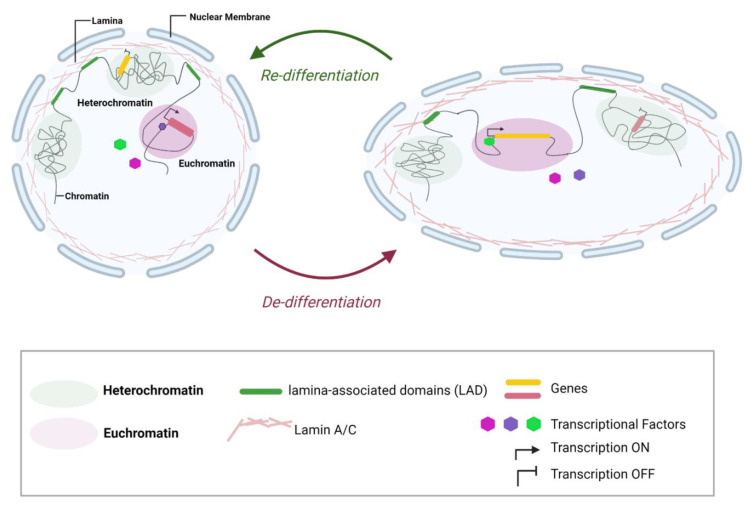
Nuclear morphological and compartmentalization changes during de-differentiation: Changes in chondrocytes nuclear morphology, chromatin compaction and genes location during de-differentiation-monolayer expansion. The morphological changes induced by the extended nuclear expansion strain, alters the organization of genes internally. Unfolding chromatin and translocating genes from area with low transcriptional factor access, to areas with high transcriptional access and vice versa ultimately affecting gene expression profiles during monolayer expansion. Created with BioRender.com (accessed on 18 September 2022).

To date, the ACI procedure has varied tissue outcomes (~30% fibrocartilage, ~48% fibro-hyaline, and ~22% hyaline cartilage) [112]. The goal in the ACI field is focused on priming the chondrocyte phenotype, through the transfer of the chondrocytes to a 3D matrix (to mimic its native niche). Many studies have suggested that the de-differentiation of chondrocytes currently seen in ACI expansion cultures is linked to structural changes that result from chondrocyte adaptation to tissue culture plastic and loss of their three dimensional constraints [47,48,49,60]. However, this has not been directly examined and needs a better understanding of chondrocyte changes in biophysical parameters such as nuclear geometrics, chromatin organization and their implications on gene expression profiles. Studies have shown there are clear nuclear structural and compartmentalization differences between heart and liver nuclei and how this affects tissue-specific-gene expression profiles [6]. To date, we do not have a clear understanding of changes that occur to the genome profile during in-vitro expansions; new technologies as MNase-seq, DNase-seq, FAIRE-seq and ATAC-seq, could provide a comprehensive map of these changes. By characterizing nuclear changes, heterochromatin alterations, and gene expression profiles in chondrocytes will allow us to identify the physical aspects that prime an irreversible de-differentiation, and presents the potential of manipulating the nuclear organization and mechanics to encourage a chondrogenic phenotype (Figure 2).

## Figures and Tables

**Table 1:** The 1st generation commercialized expansion treatments for ACI: number of cells provided for implantation procedure by the commercial provider, expansion methodology and cell density recommended for the ACI procedure.

Commercial Name (Company)	No. of Cells Provided	Expansion	Recommended Cell Implantation Density
First ACI procedure by Brittberg and Peterson	2.5–5 million cells	Monolayer, Flask	-
Carticel (Genzyme)	12 million per vial	-	≥2 × 10^6^/cm^2^ defect ≤ 7 cm^2^
ChondroCelect (TiGenix)	4 million cells per vial	Monolayer, Flask	0.8–1 × 10^6^/cm^2^
Chondrosphere Or Spherox (Co.don)	200 microliter of isotonic sodium chloride, there are 60 spheroids. (single spheroid contains 200,000 cells) 12 million cells implanted in total	Initially expanded in Monolayer, then transferred into a suspension culture	10–70 spheroids/cm^2^
